# On‐Site Evaluation of Electropositive Filtration for Fouling Control: Case Study in a Water Reuse Desalination System

**DOI:** 10.1002/wer.70541

**Published:** 2026-08-18

**Authors:** Marion Bellier, Shahnawaz Sinha, Raymond Knispel, Gary Battenberg, François Perreault

**Affiliations:** ^1^ School of Sustainable Engineering and the Built Environment, Nanosystems Engineering Research Center for Nanotechnology Enabled Water Treatment Arizona State University Tempe Arizona USA; ^2^ Argonide Corporation Sanford Florida USA; ^3^ Department of Chemistry University of Quebec in Montreal Montreal Quebec Canada

**Keywords:** carbon filter, electropositive filter, filters, fouling, pre‐treatment, reverse osmosis, water reclamation

## Abstract

Cartridge filters are used as a prefilter to reverse osmosis (RO) membranes to reduce membrane fouling during operation. However, cartridge filters typically serve as a physical barrier for foulant removal. This study reports a side‐by‐side comparison of an electropositive filtration (EPF) cartridge (NanoCeram) with a conventional string‐wound filtration (SWF) under full‐scale operating conditions at the Scottsdale Water Reclamation Facility (Scottsdale Water Campus). A customized two‐stage RO test bed operating at 500 gal per day and 25%–40% recovery was used to evaluate the performance of EPF over an 8‐week period. Performance metrics included RO permeate flux stability, membrane fouling analysis, and specific energy consumption of water production. The EPF filter was found to reduce membrane fouling, resulting in a ~20% increase in RO permeate flux and 13%–18% decrease in energy consumption in the unit operating with the EPF compared to the one using the conventional SWF. In addition, the presence of a carbon filter prior to the EPF filter was found not to be needed for its good operation. This study is the first report of an on‐site trial of EPF cartridge filters for RO fouling mitigation in a wastewater reuse setting.

## Summary

- Electropositive filtration was compared to string‐wound filter in a water reclamation facility.
- Electropositive filtration maintained the RO permeate flux ~20% higher over an 8‐week period.
- Membrane autopsy revealed less fouling accumulated on the membrane.
- Energy consumption at the RO stage was 13%–18% lower when using electropositive filtration.

## Graphical abstract

A side‐by‐side comparison was made of electropositive filtration with string‐wound filtration as prefilters to reverse osmosis in a water reuse system. The pilot system ran for 8 weeks using a side stream of a full‐scale water reuse facility in Arizona.

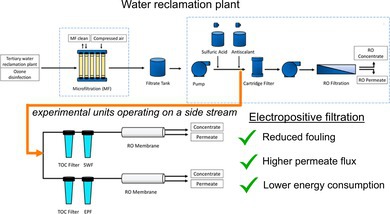

## 1 Introduction

Membrane fouling remains one of the most critical performance‐limiting factors in pressure‐driven reverse osmosis (RO) membrane processes. Fouling reduces membrane permeability, lowers permeate quality, increases transmembrane pressure, and significantly elevates energy consumption and operating costs (Ahmed et al. 2023; Guo et al. 2012; Jiang et al. 2017). Over time, fouling also shortens membrane lifespan, thereby increasing the frequency of chemical cleaning and replacement. The mechanisms of fouling are multifaceted: In natural and engineered waters, organic macromolecules, inorganic precipitates, colloidal matter, and microbial communities, all contribute to the accumulation of deposits on the membrane surface (Ahmed et al. 2023).

Pre‐treatment of feed water is therefore a critical step in both water reuse and desalination facilities. Conventional pre‐treatment processes aim to reduce the concentration of fouling constituents before they reach the RO stage (Khan et al. 2014). However, no pre‐treatment method can fully eliminate all potential foulants. The efficacy of pre‐treatment is strongly influenced by both the nature of the treatment process and the feed water composition (Abushaban et al. 2021; Brehant et al. 2002; Löwenberg et al. 2015). As a result, residual organics, colloids, or microorganisms invariably pass through, and fouling accumulates progressively during membrane operation.

The composition of feed water in wastewater reuse facilities poses unique challenges for fouling control. Elevated concentrations of dissolved organic carbon (DOC), microbial species, and sparingly soluble inorganic salts contribute simultaneously to organic, biofouling, and scaling phenomena (Chen et al. 2024; Park et al. 2024; Quay et al. 2018). Fouling is also spatially heterogeneous within an RO module: the inlet section is typically dominated by organic and biological fouling, whereas the tail‐end—where salts are concentrated by the separation process—is more prone to inorganic scaling (Abd El Aleem et al. 1998; Khan et al. 2014). Although scaling can often be controlled through the addition of antiscalants, organic and biological fouling remain considerably more difficult to manage and require advanced or alternative pre‐treatment strategies (Nunes 2020).

Electropositive filters (EPFs), such as the NanoCeram, represent a promising pre‐treatment technology to target the reduction of organic and biological foulants from feed waters. EPFs function through an electrostatic adsorption mechanism, enabling high‐efficiency removal of negatively charged soluble or particulate components without the significant pressure drop commonly associated with comparable separation processes, which preserves the water flux through the filter (Choi et al. 2023; Tepper et al. 2005). Using EPF, the removal of bacteria, viruses, oil emulsions, colloidal oxides, organic dyes, polymeric particles, and extracellular polymeric substances has been shown in synthetic solutions, tap water, surface water, or activated sludge effluents (Ikner et al. 2011; Karim et al. 2009; Miao et al. 2012; Mowers and Komlenic 2013; Soto‐Beltran et al. 2013; Tepper 2008). For membrane fouling mitigation, positive results were obtained using EPF pre‐treatment in seawater desalination and industrial water treatment applications (Bennett 2010; El‐Azizi et al. 2011; Komlenic et al. 2013). However, EPF pre‐treatment has not been reported yet for fouling mitigation in a wastewater reuse desalination scenario, where the foulants composition may differ.

To address this gap, the present study conducted a side‐by‐side comparison of NanoCeram EPF filtration and conventional string‐wound filtration (SWF) under full‐scale operating conditions at the Scottsdale Water Reclamation Facility (Scottsdale Water Campus). A customized two‐stage RO test bed operating at 500 gal per day (GPD) and 25%–40% recovery was used to evaluate the performance of EPF over an 8‐week period. Performance metrics included feed water quality, RO permeate flux stability, and specific energy consumption (SEC) of water production. Membrane autopsies were also carried out to directly assess the extent and nature of fouling with and without EPF pre‐treatment. The findings contribute to understanding the potential role of EPF in enhancing the efficiency, reliability, and cost‐effectiveness of advanced wastewater reuse desalination systems.

## 2 Materials and Methods

### 2.1 Test Site

**FIGURE 1:**
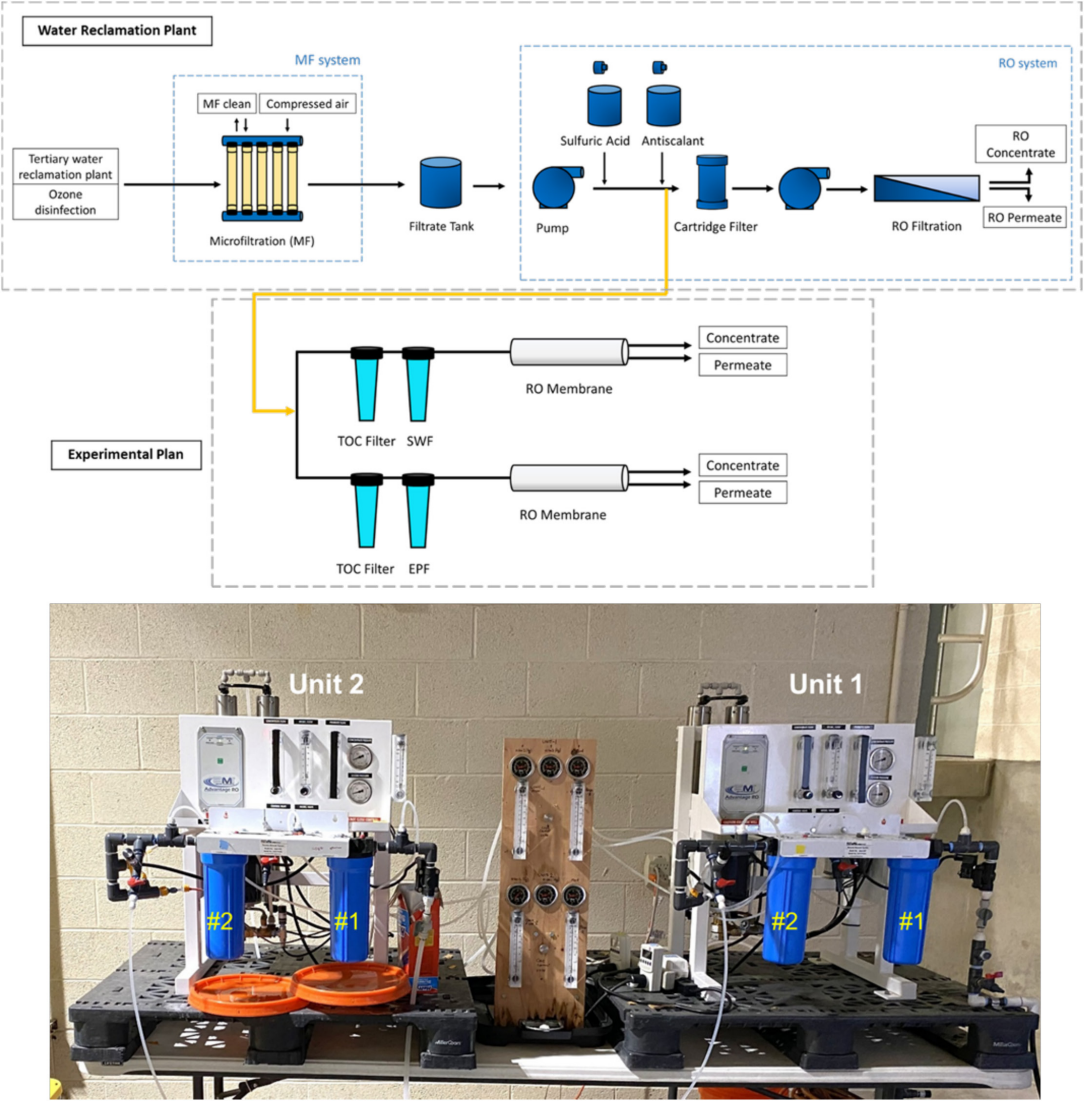
Simplified schematics of the Scottsdale Water Campus utility process with the position of our reverse osmosis system. Each unit possesses two filter housings (EPF or SWF) and two membrane modules each. A series of pressure and flow meters was added throughout the setup for remote monitoring and additional readings of the pressure and flow rate values.

The two units were installed at the Scottsdale Water Campus location. The built system was connected to a side stream of the RO feed, as pictured in Figure 1. The RO feed is a tertiary‐treated municipal wastewater effluent that has undergone conventional biological treatment, including nitrification/denitrification, as well as ozonation and microfiltration. Sulfuric acid, antiscalants, and chloramines are added to control the pH and reduce inorganic scaling during RO treatment before our side‐stream collection point. The incoming feed water had a typical pH of 6.1, a temperature of 26°C, a conductivity of 1925 μS/cm, and a DOC of 14.5 mg/L.

### 2.2 Test System

Two identical RO units (Model number P‐225A‐116; Applied Membrane Incorporated, CA) were used in parallel. The units were connected to a side stream of the RO feed of the Scottsdale Water Campus RO system and were preceded by a booster pump (PNC‐10, AMI, 0.5 HP) to maintain the pressure at the level needed for the RO units. Each unit operated at a 500 GPD capacity (110 V/1Ph/60 Hz) with two membrane elements (2.5″ × 21″, M‐T2521ALE) each. Typical operating conditions had a feed pressure of 47 psi and a feed flow of 1.5 LPM, resulting in a 35% recovery with a permeate flow rate of 0.52 LPM. The typical salt rejection was 95% and a recirculation rate of 3.4 LPM was used.

### 2.3 Water Quality Analysis

Both feed (bypass line) and treated water from these trains (i.e., after CF and after RO treatment) were sampled. Field measurements were made using handheld meters to measure pH, temperature, conductivity, and turbidity. Sampling and measurements were collected two to three times a week. Analyses of UV_254_ absorption and DOC were carried out on selected samples throughout the experiments. In addition, pressure sensors were placed on the RO units to continuously measure the feed pressure (after the booster pump, before the cartridge filters), filter inlet and outlet pressure, RO feed pressure, and concentrate pressure.

### 2.4 Membrane Fouling Analysis

Membrane modules were collected after each major experimental condition change and stored at 4°C until analysis. For analysis, modules were opened and coupons were dried at room temperature. Fouling morphology analysis was done by scanning electron microscopy (SEM) (ESEM‐FEG XL‐30, Philips Hitachi SU‐70, Hillsboro, OR) at an acceleration voltage of 15 kV. The SEM samples (1 cm^2^) were sputter‐coated with gold and platinum before SEM measurements. Membrane fouling was also characterized by Fourier transform infrared spectroscopy (FTIR) on a Bruker IFS66 V/S equipped with an MCT detector and a KBr beam splitter with a Diamond Attenuated Total Reflectance (ATR) module. A total of 32 scans were taken per sample, and all measurements were done in triplicates.

### 2.5 SEC

Two different approaches were used to determine the SEC of the test bed system. The two approaches represent the energy consumption at the membrane stage and at the system level.

For the membrane specific energy consumption (*SEC*_*membrane*_), the energy consumption per volume of permeate was calculated according to the following equation (Zhu et al. 2009):

(1) $${\mathrm{SEC}}_{\text{membrane}}=\frac{Q_{f}\left(P_{f}-P_{p}\right)}{Q_{P}}=\frac{P_{F}-P_{P}}{\mathrm{RR}}$$

where *SEC*_*membrane*_ is the specific energy consumption at the membrane level (kWh/m^3^), *RR* is recovery ratio (%), *Q*_*F*_ is feed flow rate (m^3^/h), *P*_*F*_ is feed side pressure (m), *P*_*P*_ is permeate side pressure (m), and *Q*_*P*_ is permeate flow rate (m^3^/h). It should be noted that, based on the design of the units used, the permeate side was open to the atmosphere and not pressurized. Therefore, *P*_*P*_ is 0 for our conditions. The second approach considers the energy consumption of the test bed system, termed *SEC*_*system*_. The system includes one booster pump (PNC‐10, AMI, 0.5 HP) followed by the two units installed in parallel. The energy input from the feed water from Scottsdale was not considered. For this approach, the energy consumption for each element (E_pump_ or E_unit_, in kWh, for the booster pump and RO units, respectively) was calculated by multiplying the power rating of the equipment (booster pump or RO unit, in W) with the time of operation (h), divided by the cumulative permeate volume (m^3^) for each time point, based on Equation ((2):

(2) $${\mathrm{SEC}}_{\text{system}}=\frac{E_{\text{pump}}+E_{\text{unit}}}{V_{P}}$$

The cumulative permeate volume was calculated by considering the area under the curve of the flow rate over time to include variations in permeate flux over the course of the experiment. Energy losses due to friction, leaks, or pump inefficiencies were not considered.

### 2.6 Experimental Schedule

The two sets of experiments were carried out for a total of 115 days. The first set of experiments (no C280 filter) tested the string‐wound filter (SWF) inserted in housing #2 whereas housing #1 was kept empty in the first unit. The second unit housed the EPF in housing #2 with an empty housing #1. The first experimental set lasted 59 days. The membranes were collected and replaced with new ones before the start of the next set of experiments comparing the SWF and EPF, this time with the carbon C280 filter preceding both the SWF and EPF filter. The second set of experiments (with C280 filter) spanned over the course of 56 days, testing the C280 filter (housing #1) preceding the string‐wound filter (housing #2) in Unit 1, whereas Unit 2 tested the C280 filter (housing #1) preceding the EPF (housing #2). Membrane sets were collected and analyzed for fouling analysis.

## 3 Results

### 3.1 Side‐by‐Side Comparison of the SWR and EPF Filters

**FIGURE 2:**
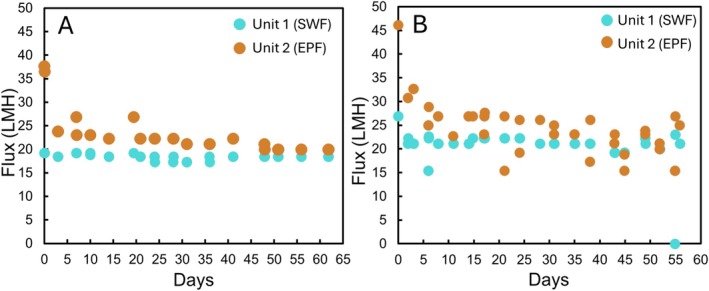
Changes in the permeate flow rate of the RO units with a SWF (Unit 1) or EPF (Unit 2), without the C280 filter (A) or with a C280 filter (B).

When the RO system performance is compared between Unit 1 and Unit 2, higher performance of Unit 2, using the EPF filter, is observed in terms of permeate flux, particularly in the absence of the carbon filter (Figure 2). In the absence of the carbon filter, the difference between the two units starts high and decreases over time as the membranes accumulate foulants. The difference in permeate flux between the two units remained observable after the 8 weeks of operation. The permeate flux of the EPF filter only was, on average, ~20%–30% higher than the permeate flux of the SWF filter only (Figure 2A). No change in SWF or EPF was needed during the 8‐week period of the experiment, as no significant headloss was observed for both units.

The comparison of the two units with the addition of a C280 carbon filter in the housing #1 of each unit is shown in Figure 2B. Although unconventional in wastewater reuse scenarios, the addition of a carbon filter before the EPF is common in other types of industrial feeds, and therefore, it was of interest to assess the performance of the RO system in the presence and absence of a carbon filter. The addition of the C280 filter as a prefilter did not result in significant changes in water flux (Figure 2B) in both units. However, it should be noted that, in the presence of the C280 filter, significant headloss was observed for both units during the duration of the experiment, which resulted in a need to change the cartridge filters. During filter changes, the membrane remained the same but the prefilters (C280 and SWF/EPF) were changed. Five filter changes were required at days 6, 11, 17, 48, and 55, when the headloss reached more than 20% of the baseline value. Filter changes happened at the same time for both units with the C280, indicating that the headloss was primarily associated with the C280 filter and not the SWF/EPF filter. This higher headloss and the need to change the filters during the experiment explains the higher variability in flux observed in Figure 2B. Therefore, the presence of the C280 filter appears to be detrimental to the operation of the unit, without observable benefits in water flux. When both conditions are compared the water flux in the units with the SWF, with or without the C280 filter, remains constant at around 20 LMH while the water flux in the units with the EPF, with or without the C280 filter, starts high in both conditions before rapidly decreasing and reaching a steady but slow decrease to approximately 25 LMH.

**FIGURE 3:**
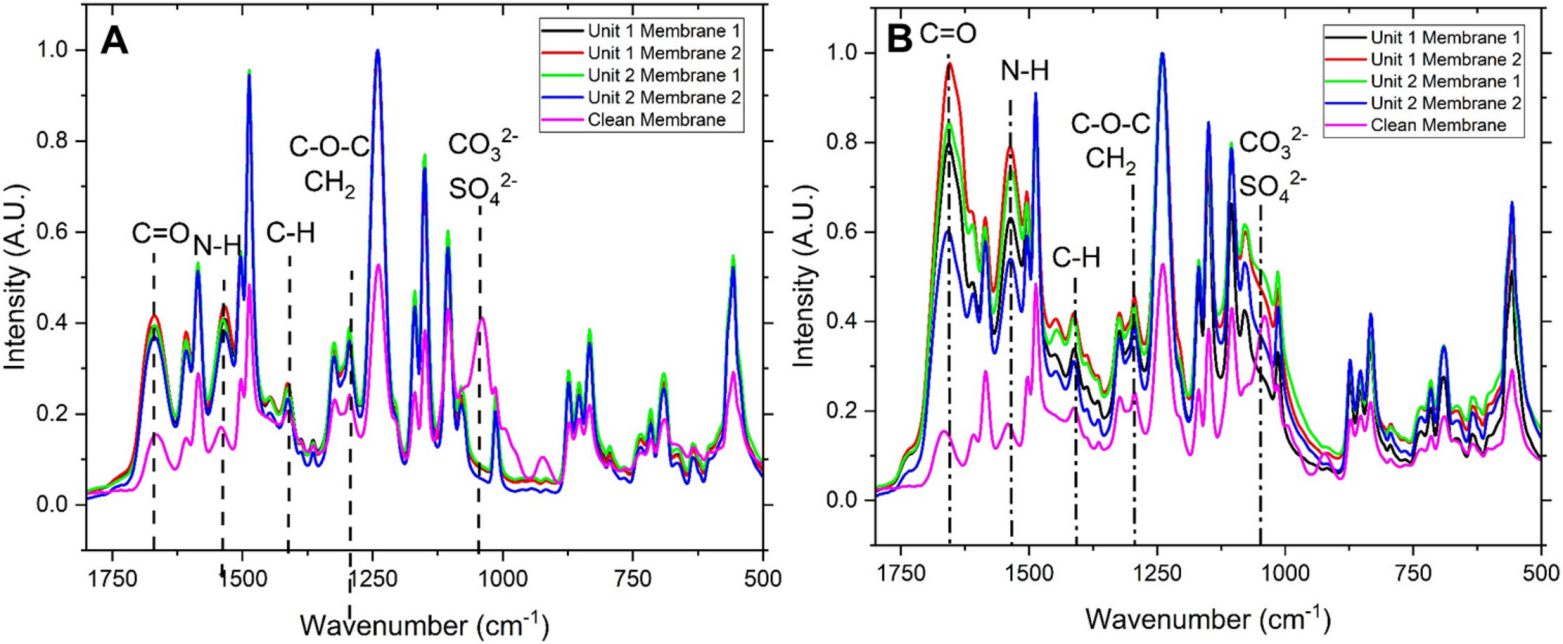
Fourier Transform Infrared (FTIR) spectroscopy of a clean polyamide TFC membrane and membranes fouled after 8 weeks of operation at the Scottsdale Water Campus. In (A), the C280 filter was not used, and the systems only contain either the SWF (Unit 1) or the EPF (Unit 2). In (B), Unit 1 is loaded with a C280 carbon filter followed with the SWF, and Unit 2 is loaded with a C280 carbon filter followed with the EPF. Coupons from both membrane elements of each unit were analyzed. Fouled membranes were collected at the end of the project and stored at 4°C until analysis.

Therefore, the EPF filter appeared to reduce fouling for the RO membrane, which maintained the permeate flux to a higher value compared to a unit operated with a conventional SWF. The presence of the C280 carbon filter appears to not be required for the EPF filter's performance; in fact, the results in Figure 2 indicate that the C280 filter decreases the EPF's capacity to mitigate fouling. To better understand the fouling behavior at the RO membrane stage, membrane fouling analyses were performed on membranes collected at the end of each experiment. The composition of the fouling layer was investigated using FTIR spectroscopy, a common approach for fouled membrane analysis (Chen et al. 2018). Figure 3 shows the FTIR spectra of a control desalination membrane (clean membrane), and coupons of the membranes taken from the first or second membrane element of Unit 1 or Unit 2. With the carbon prefilter, an important increase in the intensity of the absorbance band associated with C=O (1660 cm^−1^), C‐O‐C (1294 cm^−1^), and C‐H (~1420 cm^−1^, 1294 cm^−1^) functional groups of organic matter, as well as the amide II N‐H peak (1540 cm^−1^) associated with protein‐like compounds (Rahman et al. 2018; Zheng et al. 2018), can be observed. A broad shoulder is also observed at ~1100 cm^−1^ associated with CO_3_^2−^ and SO_4_^2−^ of inorganic fouling, as previously observed for membrane fouling generated from RO concentrate of the same feed water (Rajwade et al. 2020), but also from the polysulfone support layer of the membrane, because the FTIR signal can penetrate beyond the active layer of the membrane and into the polysulfone support of the membrane. The interpretation of this broad peak is therefore less reliable.

From the change in FTIR peak intensity compared to the clean membrane, more fouling accumulated on the membranes when the C280 filter was present (Figure 3B) than in the absence of the C280 filter (Figure 3A). In the presence of the C280 filter, the highest absorbance was observed for the C=O band of the second membrane elements of Unit 1, which was equipped with the SWF, whereas the lowest value was for Membrane 2 of Unit 2, which was equipped with the EPF. These results suggest that the C280 filter enhanced membrane fouling. This effect can be either attributed to the accumulation of organic matter in the carbon filter, which can serve as a source of microbial growth in the system and enhance fouling at the membrane level, or to the removal of chloramine, sometimes added prior to the MF stage in the plan to mitigate microbial growth in the system. Our analysis indicated a chloramine residual concentration of 3.31 mg/L in the feed water reaching our experimental units. This chloramine level was reduced to 0.87–1.52 mg/L after the carbon filter, supporting this hypothesis. Therefore, the use of a carbon filter before the cartridge filter is not recommended in a water reuse scenario.

In comparison, the FTIR spectra for the systems without the carbon filters have lower peak intensity than in Task 2, suggesting lower membrane fouling by organic or biological components. As in Task 2, the absorbance for the peaks associated with fouling, such as those for the C=O or N‐H groups, were higher for the second membrane elements of Unit 1, equipped with the SWF, whereas the lowest value was for Membrane 2 of Unit 2, equipped with the EPF. Therefore, in both experiments, the system using the EPF as a cartridge filter showed lower fouling accumulation than the system with the SWF.

**FIGURE 4:**
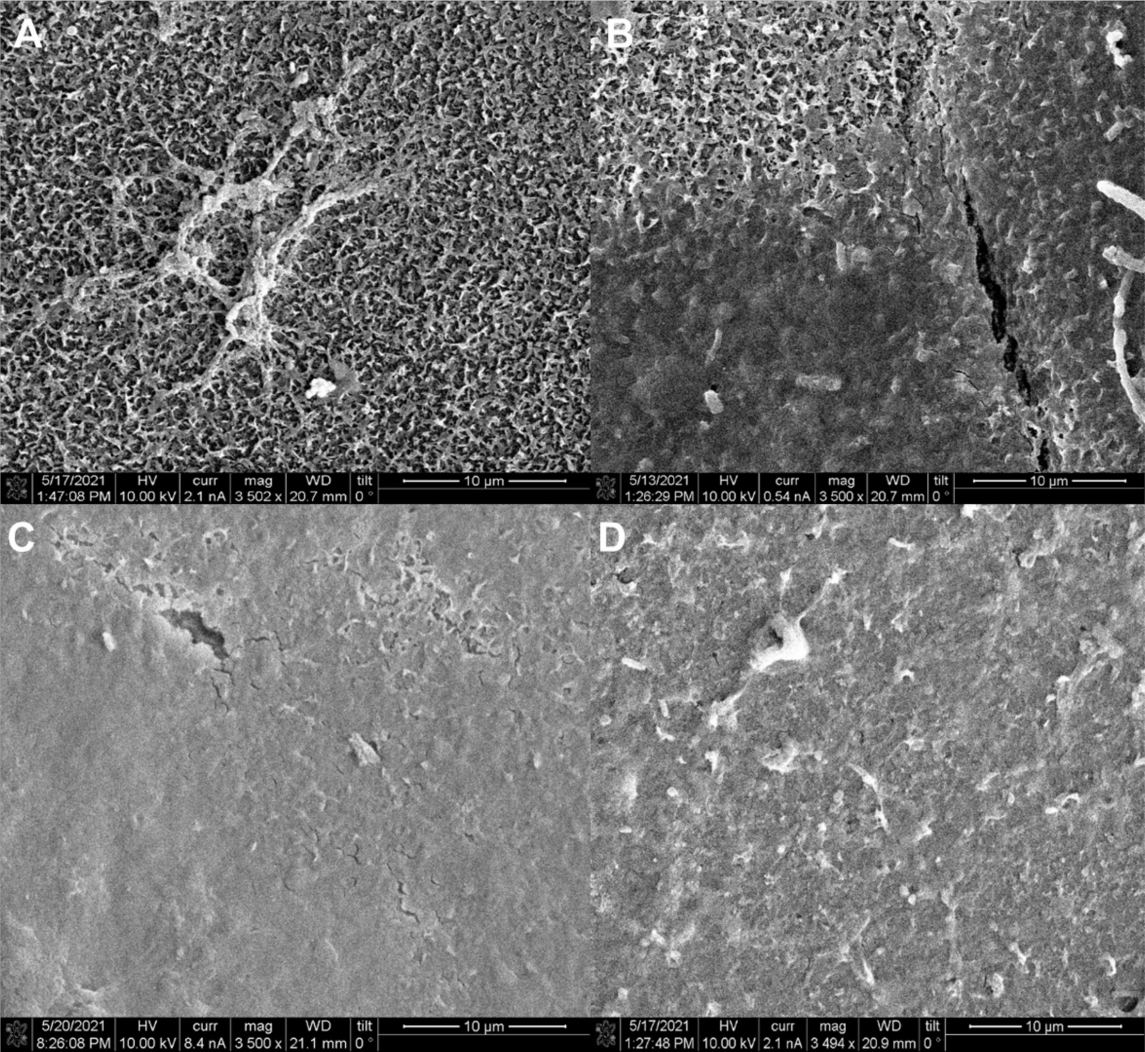
SEM micrographs of the fouled TFC membranes collected after 8 weeks of operation at the Scottsdale Water Campus. In (A) and (B), the C280 filter was not used, and the systems only contained either the SWF (A) or the NanoCeram EPF (B). In (C) and (D), the C280 filters are preceding the SWF (C) or EPF (D) filters.

The organic nature of the fouling layer on the membrane was confirmed by SEM imaging (Figure 4). The SEM micrographs of the clean TFC membrane show the characteristic ridge‐and‐valley structure of the polyamide active layer of thin‐film composite RO membranes. The fouled membranes show a dark amorphous material covering the surface but no distinctive features that would suggest inorganic (i.e., salt crystals) fouling. Some round and elongated features indicate the presence of bacteria on the surface of the membrane. Although not a quantitative analysis, when the SEM images of the membranes taken from the experiment using the C280 carbon filter (Figure 4C,D) are compared to the ones from the experiment without C280 filters (Figure 4A,B), the results support the spectroscopic results of the FTIR analyses and confirm that the C280 accelerated fouling at the RO membrane stage.

**FIGURE 5:**
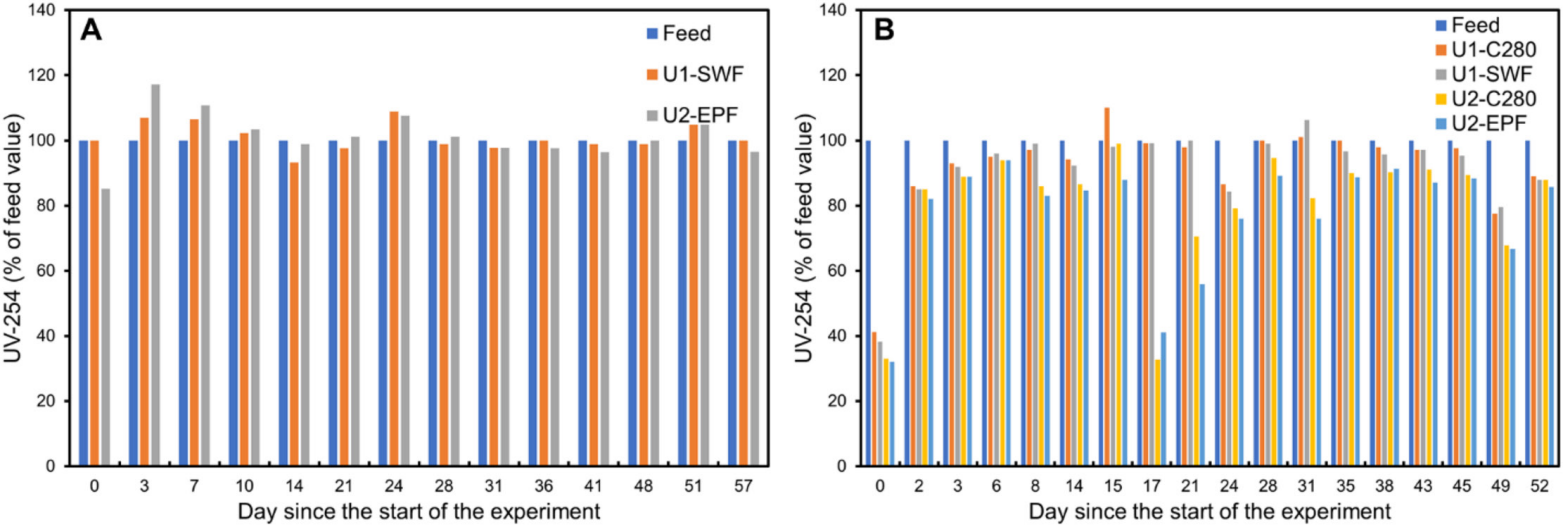
Removal of UV‐254 for the units with SWF and EPF without (A) and with (B) the presence of the C280 filter.

The removal of feed components by the different units was compared to better understand the differences observed between the two experimental designs with and without carbon filter. When the UV‐254 removal values were analyzed in the absence of C280 filters, it can be noted that neither the SWF nor the EPF filters removed significant amounts of UV254‐associated organic matter (Figure 5). Removal was higher for the unit with the EPF, but average removal for both units was < 3% and can be considered negligible (Figure 5A). On the other hand, higher DOC removal (measured at Day 25 of the experiment) was observed for Unit 2 compared to Unit 1. The DOC concentrations went from 8.335 mg/L in the RO feed to 8.627 mg/L after the SWF in Unit 1, and to 7.511 mg/L after the EPF filter in Unit 2, accounting for a 3% increase and 10.1% decrease, respectively. In the presence of the C280 filter, higher removal of both UV‐254 and DOC was observed, due to the ability of carbon filter to absorb organic matter and dissolved organic molecules from feed water. The average UV‐254 removal after C280/SWF in Unit 1 was 8.8%, whereas a removal of 22.3% was observed for Unit 2, with the C280/EPC (Figure 5B). Similarly, DOC removal (at Day 24 of the experiment) was of 16.0% and 29.3% for Unit 1 and Unit 2, respectively.

The higher removal observed for the system equipped with the EPF cartridge supports the lower membrane fouling results and the associated higher water flux for Unit 2. However, UV‐254 and DOC are only part of the different fouling components that may be encountered in the feed water, and the low removal observed for these two components by the EPF only condition may not completely explain the lower fouling observed for this system. One important component that was not measured but that was reported in the literature for EPF pre‐treatment is particulate foulants such as bacteria or transparent exopolymer particles (El‐Azizi et al. 2011; Komlenic et al. 2013). Both are associated with the development of biofilms in membrane systems (Bar‐Zeev et al. 2015), and their removal is likely to have contributed to the higher performance of Unit 2 using the EPF cartridge. In a study investigating the fouling layer composition of desalination membrane modules in a municipal wastewater reuse setting, high level of biological fouling was observed in the lead position elements of the membrane system (Khan et al. 2014), which would be the portion of the membrane system more representative of the shorter two‐module units used in this work. This observation is in agreement with the FTIR membrane autopsy analyses performed in this work. Therefore, future work aiming to better understand the underlying mechanisms by which EPF contributes to fouling mitigation in wastewater reuse conditions should further investigate the removal of this organic/biological particulate fraction, using techniques like size exclusion chromatography, resin fractionation, or fluorescence excitation‐emission matrices (Haberkamp et al. 2011; Stein et al. 2023), which would help better understand how each component contributes to the overall development of the fouling layer on the membrane after EPF pre‐treatment.

### 3.2 Effect of the Cartridge Filter on the Energy Cost for RO Desalination in the Test Bed System

**FIGURE 6:**
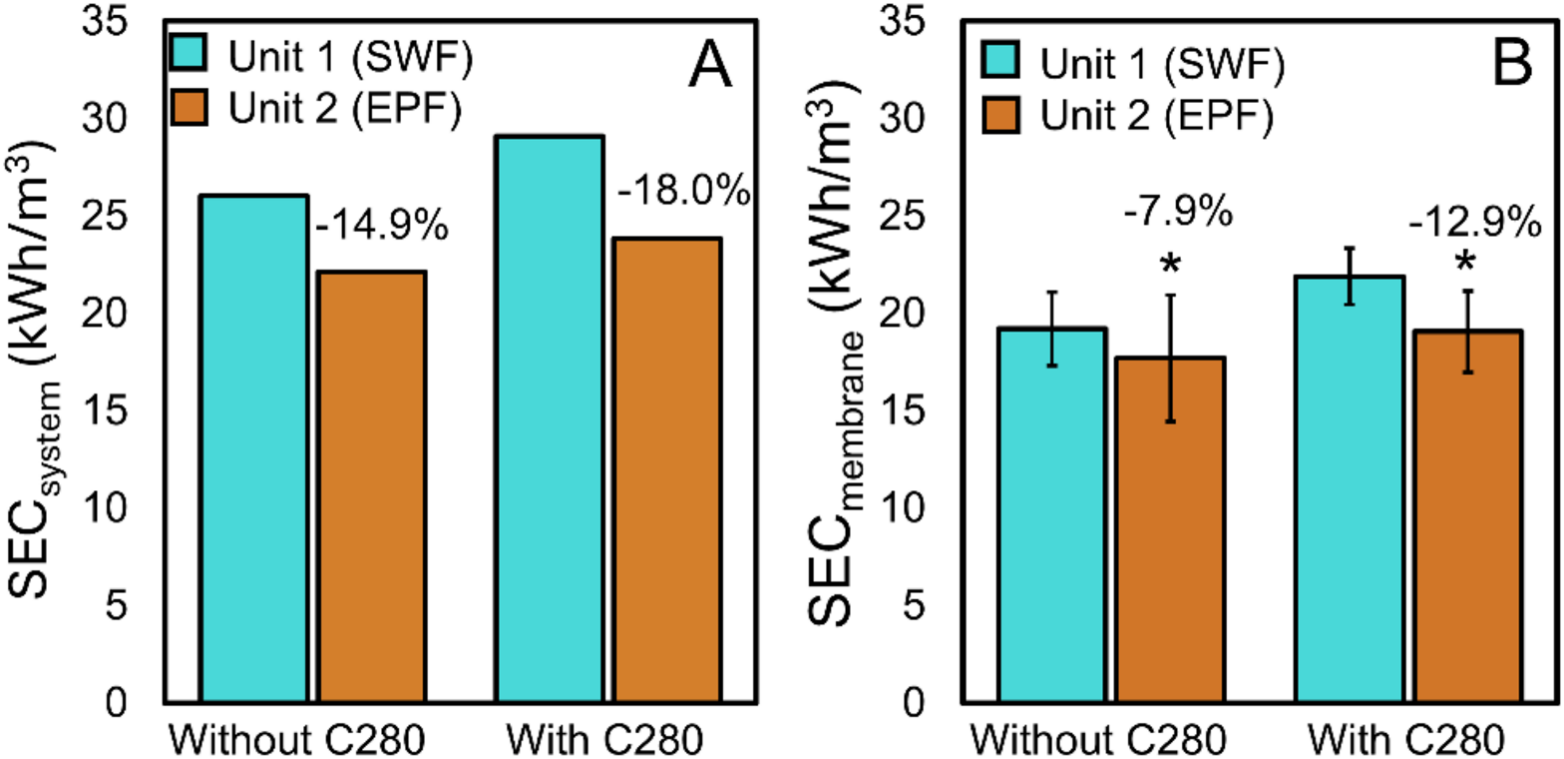
The *SEC*_*system*_ (A) and *SEC*_*membrane*_ (B) values for the two test units without and with the C280 filter. The asterisks above the bars indicate statistically different average values, based on a Student *t*‐test (*p* < 0.05). The % noted above the bar indicates the difference in SEC value between Unit 1 (SWF) and Unit 2 (EPF) for each condition (without and with C280).

The effect of the cartridge filter (SWF or EPF) on the SEC of desalination was determined to evaluate the effect of EPF filtration on the productivity of the RO system. Two approaches were considered. The first approach to determine the SEC, termed *SEC*_*system*_, considered the energy consumption of the pumps and RO systems used in the test bed systems. It does not consider the energy consumption of the pumps of the Scottsdale utility; therefore, the absolute value of this energy consumption is not accurate of the overall wastewater reuse process but only of the test bed unit. In addition, it should be mentioned that the test bed system used by our team was not optimized and that energy losses could occur at the pump level, due to friction in the pipes and to periodical leaks. However, these experimental biases would be reflected in the calculation for both units, making the comparison between Unit 1 and Unit 2 possible. The *SEC*_*system*_ of the unit tested ranged from 22 to 29 kWh/m^3^ (Figure 6A). This value is high compared to the typical values obtained for full‐scale desalination utilities (for the reason mentioned above). However, at low pressure and low recovery values, such as the conditions used in this study, SEC values of 26 kW/m^3^ have been found for small‐scale systems (Richards et al. 2007; Ruiz‐García and Nuez 2020). The benefits of the EPF are observed in both runs with and without the carbon filter. In the absence of C280 carbon filter, the EPF resulted in an average decrease in *SEC*_*system*_ for the RO system of 18%.

The effect of the NanoCeram EPF filtration on RO energy consumption at the membrane stage was evaluated using *SEC*_*membrane*_, calculated using Equation ((1) (see Section 2.5). This considers only the energy consumed for permeation at the membrane level. Changes in *SEC*_*membrane*_ indicate a fouling‐specific change in energy consumption. This calculation was based on the collected data from the online pressure sensors placed on the unit, collecting daily values of the RO feed pressure. However, a limitation in the experimental system, where the permeate was discarded and therefore unpressurized, makes the calculated *(P*_*f*_ *—P*_*p*_ *)* higher than it could be in a full‐scale system. When *SEC*_*membrane*_ is considered, a similar trend is observed, with the EPF filter having a lower SEC compared to the SWF filter in the run with and without the C280 filter (Figure 6B). The benefits of the EPF filter on the *SEC*_*membrane*_ are lower than on the *SEC*_*system*_, which suggests that other benefits of the EPF filter, such as low pressure drop, are also contributing to the system‐level performance of the EPF filter. A student *t*‐test was performed for the average *SEC*_*membrane*_ value throughout the duration of the task between Unit 1 and Unit 2. The difference in membrane‐level SEC is found to be statistically significant (*p* < 0.05) for both Tasks 1 and 2 (Figure 6B).

**FIGURE 7:**
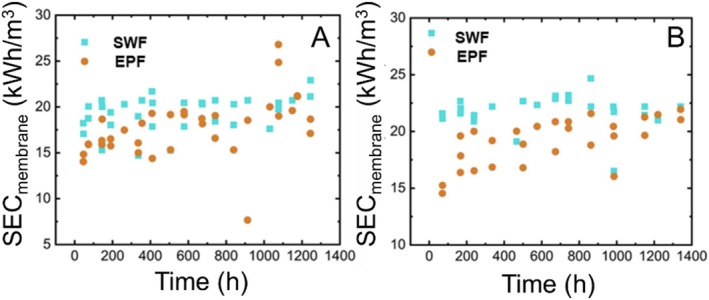
Change in the *SEC*_*membrane*_ for both filters, without (A) and with (B) the C280 prefilter*.*

Using the *SEC*_*membrane*_ parameter, the change in energy consumption over the course of the experiment can also be observed. The visualization of the *SEC*_*membrane*_ over time allows us to determine the effect of fouling on the *SEC*_*membrane*_, which is seen by the gradual increase in *SEC*_*membrane*_. The disparity between Unit 1 (with SWF) and Unit 2 (with EPF) is the most pronounced in the experiment without the C280 filter (Figure 7). This pattern correlates with the average *SEC*_*membrane*_ value of Figure 6B and the lower membrane fouling observed in Figure 2.

## 4 Conclusion

This study evaluated the performance of the EPF filter compared to a conventional SWF cartridge filter using test bed units installed at the Scottsdale Water Campus. A side‐by‐side comparison was done on site using a side stream of the RO feed water. The following conclusions can be obtained from this study:

- The EPF filter reduced membrane fouling compared to the conventional SWF over an 8‐week period.
- The EPF filter increased RO permeate flux compared to the SWF. This effect diminished throughout the duration of the experiments; however, the unit operated with the EPF maintained higher flux, with an average of ~20% improved performance compared to the unit operated with a SWF.
- The use of an EPF filter reduced by 13%–18% the energy consumption at the system level (*SEC*_*system*_) for RO desalination compared to the unit with a conventional SWF.
- The presence of a carbon filter is not needed for good operation of the EPF filter in a wastewater reuse setting. In fact, the presence of a carbon filter increased membrane fouling, required frequent filter changes, and reduced the overall benefit of the EPF filter.

Therefore, based on the reduced energy consumption, EPF filters used as cartridge filters before the RO unit can benefit RO desalination for water reuse. Membrane autopsy and fouling analysis suggest that this benefit of the EPF cartridge may be attributed to a reduction of fouling at the membrane level, particularly organic and biological fouling. This interpretation is in line with previous observations of EPF filters being able to remove particulate membrane fouling precursors such as microorganisms and transparent exopolymer particles (El‐Azizi et al. 2011; Komlenic et al. 2013).

The limitations of this work should be acknowledged, as the small‐scale RO systems used in this study only partially reproduce the fouling behavior of a full‐scale municipal wastewater reuse desalination system. The small module size resulted in a low (25%–40%) water recovery in the experimental units compared to the ~85% recovery in the full‐scale plant. This difference in recovery will change the fouling patterns in the membrane modules. Indeed, a membrane autopsy study realized for a full‐scale municipal wastewater reuse system showed that lead membrane elements are characterized by a higher accumulation of organic and biological foulants, whereas tail elements, on the other hand, typically show higher levels of inorganic foulants due to the progressive concentration of inorganic species that are rejected from the feed by the RO membrane (Khan et al. 2014). This inorganic fouling portion was therefore not captured in our system. In addition, the SEC values presented in this work are not representative of the SEC values in the full‐scale system. Indeed, for the SEC parameters (*SEC*_*system*_ and *SEC*_*membrane*_) calculated for the experimental units, the values do not consider energy consumption prior to the RO units (secondary wastewater treatment, feed pre‐treatment, and feed pressure), the units did not possess an energy recovery device, and the permeate stream was unpressurized. Therefore, if our results do indicate lower energy consumption for the unit using the EPF cartridge, the relative benefits at the full‐scale level may be different when a more optimized system is considered.

Given these considerations, future work investigating the potential applications of EPF pre‐treatment for fouling mitigation in municipal wastewater reuse would benefit from scaling up the study system to be more representative of a full‐scale plant in terms of energy balance and water recovery. This would allow for a better representation of the potential energy savings that can result from the higher fouling prevention associated with EPF pre‐treatment. A longer duration experiment with replicated units would also be essential to reliably capture the longer term implications of using EPF cartridges instead of SWF, such as those associated with filter variability, filter replacement, membrane cleaning frequencies, cleaning efficiency, and membrane operational lifespan. These considerations will be essential to quantify the true cost‐benefits and economic viability of EPF as pre‐treatment systems in municipal wastewater reuse desalination.

## Author Contributions

**Marion Bellier:** conceptualization, data curation, formal analysis, investigation, methodology, validation, visualization, writing – original draft. **Shahnawaz Sinha:** conceptualization, data curation, formal analysis, funding acquisition, investigation, methodology, project administration, resources, supervision, validation, visualization, writing – review and editing. **Raymond Knispel:** conceptualization, funding acquisition, methodology, project administration, resources, writing – review and editing. **Gary Battenberg:** conceptualization, funding acquisition, methodology, project administration, resources, writing – review and editing. **François Perreault:** conceptualization, funding acquisition, methodology, project administration, resources, supervision, validation, visualization, writing – review and editing.

## Funding

This work was funded by the Argonide Corporation in collaboration with the National Science Foundation Nanosystems Engineering Research Center on Nanotechnology Enabled Water Treatment (EEC‐1449500). M.B. acknowledges the support of a NSF Graduate Research Fellowship Program award (#2020307949). We acknowledge the use of facilities within the Eyring Materials Center at Arizona State University supported in part by NNCI‐ECCS‐2025490.

## Conflicts of Interest

This work was funded in part by Argonide Corporation, which developed and commercializes the NanoCeram filters. R.K. and G.B. are currently employed by Argonide Corporation.

## Data Availability

Data will be made available upon request.
